# Extracellular vesicles from focal segmental glomerulosclerosis pediatric patients induce STAT3 activation and mesangial cell proliferation

**DOI:** 10.1371/journal.pone.0274598

**Published:** 2022-11-14

**Authors:** Naile T. Pekkucuksen, Lauren P. Liu, Rasha Aly, Lawrence R. Shoemaker, Abdel A. Alli

**Affiliations:** 1 Division Pediatric Nephrology, Department of Pediatrics, University of Florida, Gainesville, Florida, United States of America; 2 Department of Physiology and Aging, University of Florida, Gainesville, Florida, United States of America; 3 Department of Medicine Division of Nephrology, Hypertension, and Renal Transplantation, University of Florida College of Medicine, Gainesville, Florida, United States of America; Center for Molecular Biotechnology, ITALY

## Abstract

**Introduction:**

Primary focal segmental glomerulosclerosis (FSGS), a major cause of end-stage kidney disease (ESKD) in adolescents and young adults, is attributable to recognized genetic mutations in a minority of cases. For the majority with idiopathic primary FSGS, the cause of the disease is unknown. We hypothesize that extracellular vesicle (EVs), that carry information between podocytes and mesangial cells, may play a key role in disease progression.

**Material & methods:**

A total of 30 participants (20 primary nephrotic syndrome/ 10 healthy controls) were enrolled in this study. Primary nephrotic syndrome subjects were grouped based on pathologic diagnosis. The FSGS group was compared to healthy control subjects based on demographic and clinical findings. EVs were isolated from the urine of each group before being characterized by Western blotting, transmission electron microscopy, and nanoparticle tracking analysis. The effects of the EVs from each group on normal human mesangial cells and activation of certain pathways were then investigated.

**Results:**

Based on demographic and clinical findings, mean serum creatinine was significantly higher in the FSGS group than the normal healthy control group. The mean size of the EVs in the FSGS group was significantly higher than the healthy control group. The mesangial cells that were challenged with EVs isolated from FSGS patients showed significant upregulation of STAT-3, PCNA, Ki67, and cell proliferation.

**Discussion:**

Our data demonstrate that EVs from FSGS patients stimulate mesangial cell proliferation in association with upregulation of the phospho-STAT-3 pathway. Additional studies are planned to identify the molecular cargo within the EVs from FSGS patients that contribute to the pathogenesis of FSGS.

## Introduction

Nephrotic syndrome (NS) refers to kidney disease associated with specific clinical and biochemical features that include: generalized edema, significant proteinuria, hypoalbuminemia, and hyperlipidemia [1,2]. NS may be secondary to systemic conditions, such as diabetes, infections, paraneoplastic, and rheumatic disorders, or it may be a primary nephropathy [1,3]. Primary childhood nephrotic syndrome is further classified by renal biopsy of children and adolescents with frequently relapsing, dependent, or resistant response to a standard course of corticosteroid therapy. Biopsies of patients with FSGS have glomerular scars in a focal and segmental distribution, as well as tubular atrophy primarily localized at the juxtaglomerular region. Mesangial proliferation may or may not be prominent in cases of FSGS [4].

Extracellular vesicles (EVs) are membrane-enclosed vesicles that carry molecules including proteins, lipids, and nucleic acids (RNAs) [5]. The main subgroups of EVs are described as exosomes, microvesicles (MVs) and apoptotic bodies based on their size, function and biogenetic pathways [5]. The origin of EVs present in the urine (uEVs) can be traced back to various kidney cell types and these vesicles have been described as rich sources of disease biomarkers [5]. Exosomes are usually smaller than 200nm in diameter and they originate from multivesicular bodies [6] Microvesicles are typically larger than 200 nm in diameter and they originate from outward budding of the cell membrane [7]. Apoptotic bodies represent the largest type of extracellular vesicles, originate from dying cells, and contain intact organelles among other cargo found in microvesicles and exosomes [6,8].

In patients with idiopathic (without a known genetic cause) primary FSGS, the podocytopathy is thought to result from interaction with systemic or a local cytopathic biochemical milieu, consistent with the Shalhoub hypothesis [9]. In glomerular visceral and parietal podocyte cell cultures and isolated glomeruli, a myriad of regulatory proteins and miRNAs, isolated from patient serum or urinary exosomes, have been found to cause cytopathic effects and /or increase glomerular permeability [10]. Primary FSGS typically presents secondary to podocyte dysfunction and is characteristic of foot process effacement with sclerosis extracellular matrix expansion and thickening of the glomerular basal membrane (GBM) that, contributes to the progression of kidney injury to end-stage kidney disease (ESKD) [11,12]. FSGS is the most common primary glomerular disease in dialysis patient in the USA [13]. The exact cause of primary FSGS is still unknown. Many different types of proteins including integrins, receptors, soluble proteins, exosomal proteins and miRNAs have been found to contribute to the pathogenesis of this disease [14–21]. These molecules were found to have an adverse effect on glomerular visceral epithelial cells such as mesangial cells and/or parietal epithelial cells and podocytes [14,22,23].

Mesangial cells are one of the main cell types that compose the glomerular apparatus. These cells interact with podocytes and endothelial cells and their dysfunction leads to various kidney diseases [24]. Mesangial cell proliferation may result from various mechanisms including the inflammatory process, medications, endothelial cell damage, and podocyte autophagy [25,26].

The signal transducer and activator of transcription (STAT) is a protein complex that is activated by multiple cytokines and growth factors [27]. This pathway regulates many cellular functions including cellular proliferation, growth, regeneration, and immune responses [27]. STAT-3 is the central member of this system that serves as a transcription factor in the nucleus [28]. It was shown that STAT-3 activation in mesangial cells alters the immune response, promotes mesangial cell proliferation and glomerulonephritis in diabetic nephropathy, human immunodeficiency virus (HIV)-associated nephropathy (HIVAN), toxic nephropathy and acute kidney injury (AKI) [29–31]. It was shown that JAK-STAT signaling is activated in the kidney and peripheral blood cells of patients with focal segmental glomerulosclerosis [28]. Although EVs can carry various signaling molecules their role in the activation of STAT-3 in mesangial cells has not been investigated.

Here we tested our hypothesis that EVs isolated from the urine of FSGS pediatric patients activate STAT-3 in normal human mesangial cells resulting in increased proliferation in these cells. To our knowledge, this is the first study investigating the role of EVs in the pathophysiology of primary FSGS in a pediatric population. Motivated by this, we provide insight into a putative mechanism by which EVs released during pediatric FSGS contribute to changes at the glomerular level.

## Material and methods

### Human subjects

This study was conducted at the University of Florida (UF), Gainesville FL between March 2020 and February 2021. The study was approved by our Institutional Review Board (IRB) [IRB201903295]. Written inform consent is obtained from legal guardian or patient if patient’s age ≥18 years old. Documentation of children’s assent were obtained according to federal regulations and UF policies.

Children who were diagnosed with primary childhood nephrotic syndrome were included in this study. Children less than 1 or greater than 21 years old, pregnant, presenting with low C3 complement level, any extra-renal disorder, systemic inflammatory disorder, or neoplastic disorder concurrent with nephrotic syndrome were excluded from the study. Demographic and clinical information including age, gender, ethnicity, height and weight, age at diagnosis of CNS and pathophysiologic type, urinalysis results and most recent plasma creatinine level, dose and frequency of prescribed medicines at time of urine collection were collected.

Random urine samples were collected from patients who were treated for childhood nephrotic syndrome (CNS) at outpatient visits as well as age and gender matched healthy controls. The urine was initially stored in a minus 20°C freezer before being transferred to a minus 80°C freezer until it was processed for EV isolation.

### Smartlyte electrolyte analysis and osmometry

The concentrations of the urinary electrolytes including sodium, potassium, and chloride, were measured using a SmartLyte analyzer (SmartLyte, Diamond Diagnostics, Holliston, MA). One ml of urine collected from each patient was centrifuged at 13,000 x g for 6 minutes. The samples were then prepared by adding 1-part urine and 2-parts urine diluent (Diamond Diagnostic) before being run on the SmartLyte machine. Urine osmolality was measured through an auto-sampling turntable model 2430 osmometer (Precisions Systems Inc; Natick, MA).

### Extracellular vesicle isolation

Extracellular vesicles were isolated from 13ml of urine obtained from each patient sample of each group. The urine was centrifuged at 450 rcf for 10 minutes at 4 degrees Celsius. The supernatant was filtered with a 0.22μm Nalgene filter (Thermo Fisher Scientific; Waltham MA) before 9ml of the filtrate was subject to ultracentrifugation at 122,653 X g (Rmin) for 90 minutes at 4 degrees Celsius using a Ti-70.1 rotor (Beckman Coulter, Brea, CA, USA). The EV pellet was resuspended in 200μl of sterile 1xPBS (Thermo Fisher Scientific) and the reconstituted EVs were characterized by multiple approaches.

### Nanoparticle tracking analysis

Both the size and concentration of the EVs were measured using an NS300 machine coupled to the NTA 3.4 Build 3.4.4 Software (Malvern; UK) at 25 degrees Celsius. The software calculates size based on the relationship between Brownian motion and hydrodynamic diameter through the Stokes-Einstein equation. NanoSight NS300 equipped with a high-sensitivity Hamamatsu sCMOS camera, ×20 objective lens, and a 50 mW green 532 nm laser and with high flow rate for the syringe pump (setting 150 μl/s). Concentration was calculated by particle observation on a frame-by-frame basis by the sCMOS camera (complementary metal oxide semiconductor; 25 frames/s). When recording the video, it averaged the concentration across all the frames, giving an absolute number average. Samples were processed across 3 × 60-s videos. A 1:1000 dilution of the EVs prepared in ultrapure 1xPBS was injected into the system and an automatic infusion pump fed the samples through the machine at an infusion rate of 65

### SDS page and Western blotting

An aliquot of the EVs was lysed in an equal volume of RIPA buffer (Thermo Fisher Scientific) before total protein concentration was determined by a bicinchoninic acid protein assay (Thermo Fisher Scientific). Fifty micrograms of total protein concentration were loaded into 4–20% Tris HCl polyacrylamide gels using the Criterion electrophoresis system (Bio-Rad). The resolved proteins were transferred onto nitrocellulose membranes (GE Healthcare, Piscataway, NJ) using the Criterion transfer system (Bio-Rad). The nitrocellulose membranes were then blocked in 5% nonfat milk 1xTBS (TBS; Bio-Rad) for 1 hour at room temperature before being washed twice with 1xTBS and incubated with primary Caveolin-1 antibody (Cell Signaling Technology; 3267), CD9 (abcam; ab223052), Syntenin antibody (abcam; ab19903), HDAC2 antibody (Cell Signaling; 2540) and Uromodulin antibody (Novus; MAB5144) at a 1:1000 dilution prepared in 5% BSA 1XTBS for at least 8 hours at 4 degrees Celsius while on a rocker. The membranes were then washed three times with 1xTBS and incubated with horseradish peroxidase (HRP)-conjugated goat anti-rabbit secondary antibody at a 1:3000 dilution prepared in blocking solution at room temperature for 1 hour. After the incubation, the membranes were washed four times in 1xTBS, incubated with Super Signal West Pico reagent (Thermo Scientific) for 7 minutes, and then imaged with a Bio-Rad imager.

### Transmission electron microscopy

Freshly isolated EV pellets were prepared in a solution of ultrapure 1xPBS and paraformaldehyde (2% final concentration). Formvar-carbon coated grids (Ladd Research Industries; Williston, VT) were placed onto 10μl drops of the EV suspensions deposited on a section of Parafilm and then allowed to absorb for 20 minutes at room temperature. The grids were transferred to 100μl drops of 1xPBS and incubated for 3 minutes. Next, the grids were transferred to 50μl drops of 1% glutaraldehyde (Ladd Research Industries) prepared in 1xPBS and then incubated for 5 minutes at room temperature. The grids were then washed in a series of eight 50μl drops of 1xPBS wash buffer for 2-minute intervals before being transferred to 50μl drops of uranyl-oxalate solution, pH 7 prepared from uranyl acetate (Ladd Research Industries) and oxalic acid (Sigma-Aldrich; St. Louis, MO) and then incubated for 5 minutes at room temperature. Thereafter, the grids were incubated in 50μl drops of methyl cellulose-uranyl-acetate and incubated for 10 minutes on a cold plate. The grids were carefully removed with a stainless-steel loop and blotted on No.1 Whatman filter paper on the grid side. Afterwards, the grids were air dried for 5–10 minutes and then viewed on a Hitachi H-7600 transmission electron microscope (Hitachi High Technologies America, Inc, Clarksburg, MD) equipped with AMT imaging software (Advanced Microscopy Techniques Corporation).

### Cell culture and EV treatment

Primary human glomerular mesangial cells (Cell Systems; Kirkland, WA) were cultured in complete classic medium with Serum and CultureBoost™ (4Z0-500) (Cell Systems). Cells were treated with 2X10^6, 2X10^7, or 2X10^8 particles/mL of EV’s from subjects who were diagnosed with FSGS, or 2X10^^7^ particles/mL of EVs from healthy human controls. The cells were scraped in RIPA Buffer containing Halt protease and phosphatase inhibitors (ThermoFisher Sci) and the cell lysate was sonicated twice for 5 second intervals while on ice. The cell lysates were than stored at minus 80 degrees Celcius. Cells with a passage number of less than 8 were used for experiments. Cells were between 90–95% confluent at the time of each experiment.

### Cell proliferation assay

A brdU cell proliferation ELISA assay (abcam; ab126556) (Waltham, MA) was performed according to the manufacture’s instructions with the following modifications. Human mesangial cells were plated at a concentration of 5,000 cells per well of a 96 well plate and cultured for 8 hours before the cells were incubated with different concentrations of EVs (2X10^6 to 2X10^8 particles/mL) for 6 hours and then treated with the BrdU reagent.

### Statistical analysis

Statistical analysis was performed using Stata version 16 (StataCorp LLC, College Station, TX), Sigma plot version 14.0 (Systat Software, San Jose, CA) GraphPad Prism 9 to determine statistical significance between various groups. Descriptive statistics were reported as percentage (%), mean ± standard deviation or median (interquartile range [IQR]) to define patient characteristics. Difference between the groups were determined by a student t-test and reported as mean +/- SEM. A Shapiro-Wilk W test was used to test normality.

## Results

### Patient characteristics

A total of 20 primary nephrotic syndrome and 10 controls who were ≤21 years old, were enrolled in the study. Two patients were excluded due to having oncologic and rheumatologic diseases. Primary nephrotic syndrome patients who were biopsied due to steroid resistance. All included study subjects were biopsied. All FSGS patients have pathological diagnosis. Only FSGSs’ and controls were analyzed. Hence all FSGS patients were under remission during the sampling and all had uPr/Cr ratio less than 0.2. Demographic and clinical characteristics are shown in Table 1. Mean age was 14.3±5 years for the FSGS patients and 9.7±4.3 years of age for the control subjects. Also, 83.3% of FSGS and 60% of controls were female. Fifty percent of the FSGS patients and 40% of the control subjects were Caucasian. Baseline urinalysis and urine chemistry findings were found to be similar in both groups. Baseline blood chemistry findings showed significantly higher mean serum creatinine levels in the FSGS patients compared to the control subjects (p<0.05).

**Table 1:** Demographic & clinical features of study population.

Characteristics	FSGS (n = 6) Mean (± SD)	Healthy Controls (n = 10) Mean (± SD)
**Gender(n = F)**	5 (83.3%)	6 (60%)
**Race (others/AA/ Caucasian)**	1/2/3	4/2/4
**Age (mean)**	14.3 (5.0)	9.7 (4.3)
**Urine Ph (mean)**	6.58 (0.74)	6.125 (0.85)
**Urine specific gravity**	1019.17 (7.11)	1019.75 (8.77)
**Urine Osm**	703 (465.99)	719 (352.39)
**Urine Sodium**	104.83 (108.22)	140.6 (32.33)
**Urine Potassium**	28.88 (25.9)	23.95 (17.20)
**Urine Chloride**	131.2 (112.8)	131.5 (38.46)
**Serum Creatinine, mg/dl**	1.25 (1.56)	0.49(0.18) *Δ
**Serum Albumin, g/dl**	4.05 (0.32)	4.27(0.25) *
**Serum Sodium, mmol/L**	138.33 (1.03)	139.2(1.79) *
**Serum Potassium. mmol/L**	3.78 (0.39)	4.06(0.49) *
**Serum Chloride, mmol/L**	105.33 (3.33)	103.2(1.92) *

* Covers only 5/10 controls lab results.

Δ Significantly lower in controls.

### Urine osmolality and electrolyte analysis for FSGS patients and control subjects

**Fig 1:**
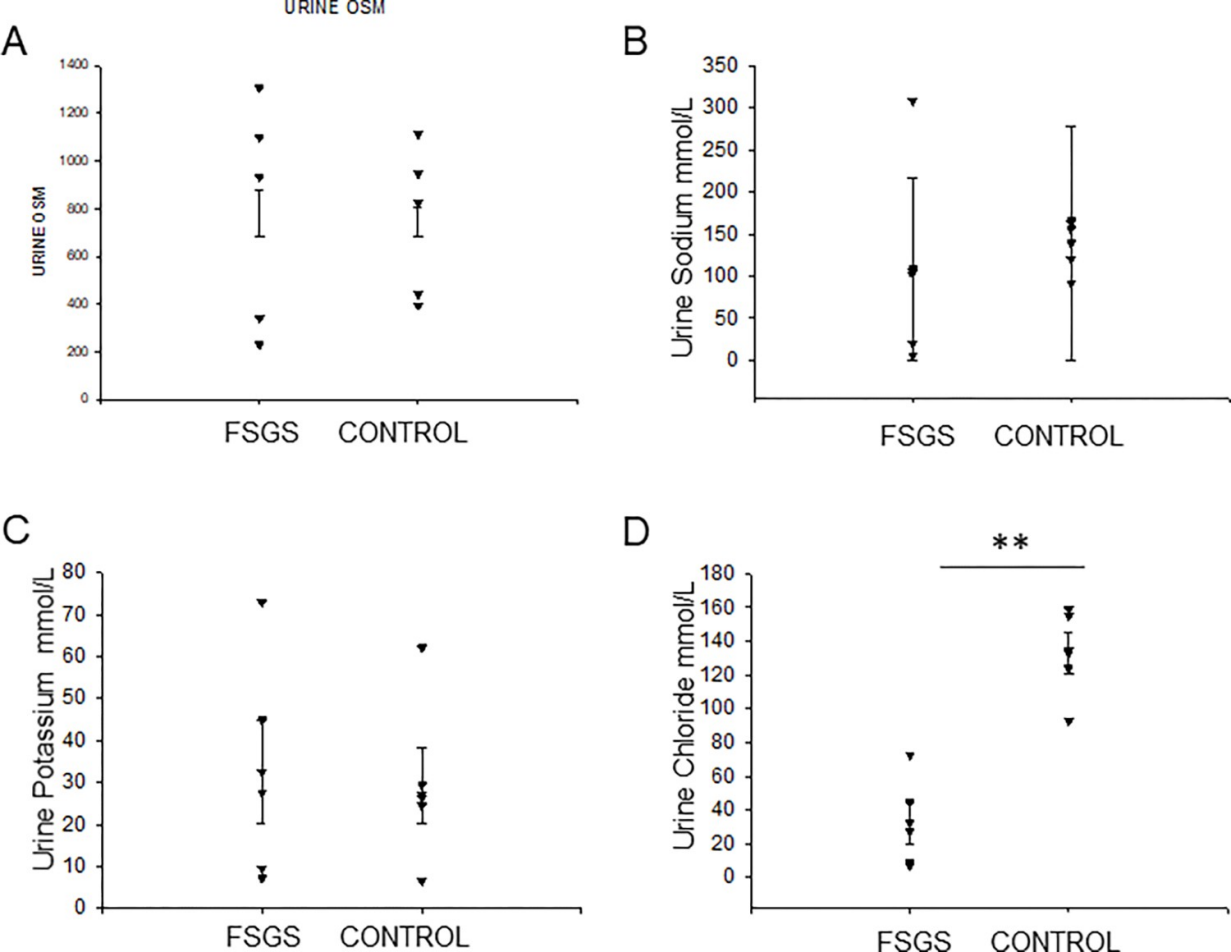
Urine osmolality and electrolyte analysis of samples from FSGS pediatric patients and age-matched healthy control subjects. Spot urine collections from FSGS patients and healthy control subjects were used to measure urine osmolality **(A)**, urinary sodium **(B)**, urinary potassium **(C)**, and urinary chloride **(D)** concentrations. SigmaPlot software Version 14.0 was used to determine statistical significance between the two groups. ** Represents p<0.001.

The concentrations of the urinary electrolytes including sodium, potassium, and chloride, and urine osmolality was important to show that all subjects had intact tubular function and good concentrating ability. After analyzing all subjects’ (N = 16) urine samples, we compared FSGS(N = 6) vs controls(N = 10). The mean urine osmolarity was found to be similar for both groups (Fig 1A). The mean urinary sodium and potassium levels were similar, as well (Fig 1B and 1C), However, we found lower mean urinary chloride levels in the FSGS group compared to the control group (Fig 1D).

### Characterization of freshly isolated EVs from FSGS patients and control subjects

**Fig 2:**
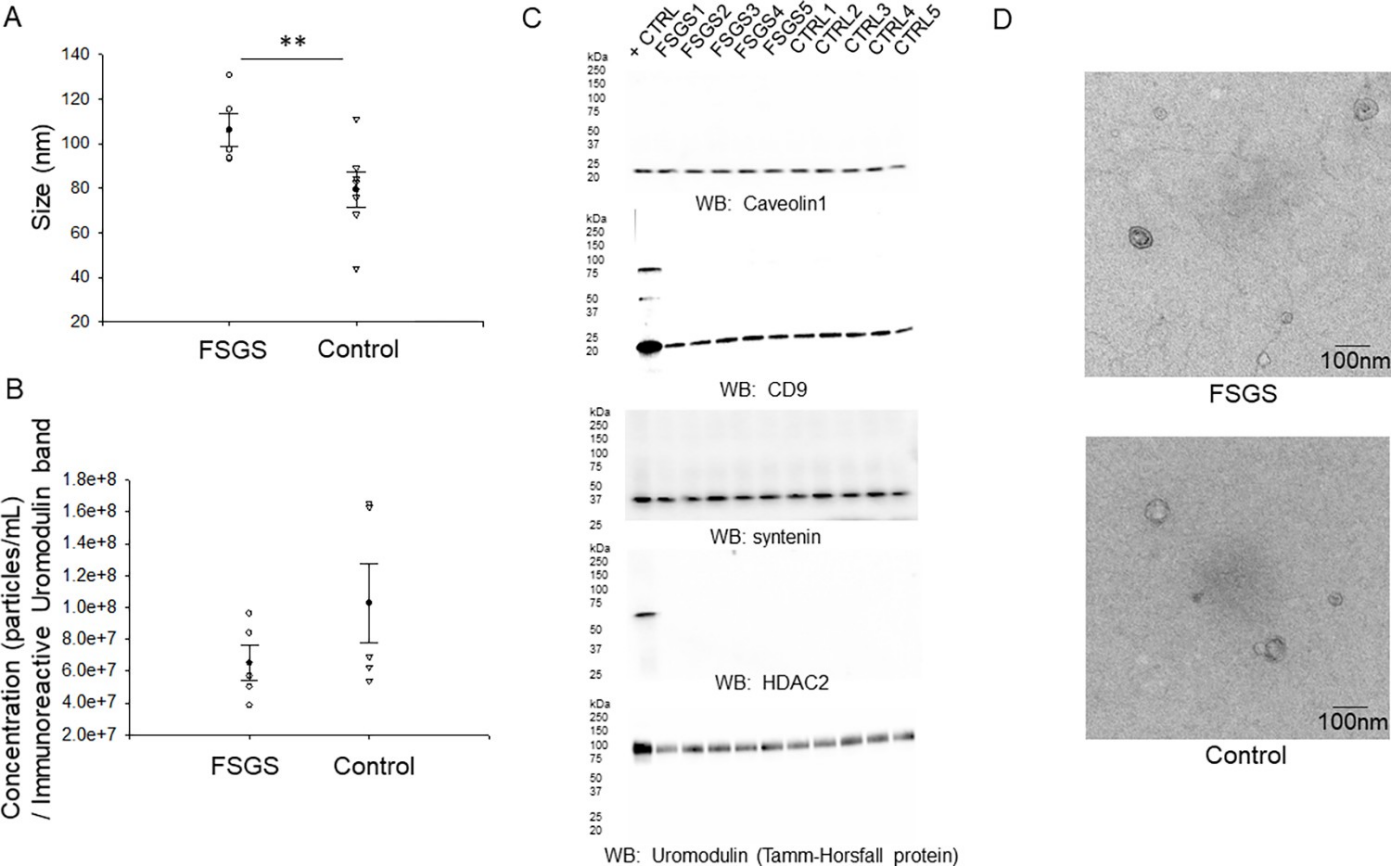
Characterization of EV from FSGS patients and control subjects. **A.** Summary scatter plot of nanoparticle tracking analysis showing EV size (N = 5 for FSGS group and N = 7 for control group). **B.** Summary scatter plot of nanoparticle tracking analysis showing EV concentration (N = 5 for FSGS group and N = 5 for control group). **C.** Western blot analysis of the EV markers Caveolin-1, CD9, and Syntenin. Each EV preparation from the two groups was probed for the non-EV negative control marker HDAC2 and Uromodulin (Tamm-Horsfall protein). The positive control (+ CTRL) lane included cell lysate from the human kidney proximal tubule epithelial cell line HK2. **D.** Representative electron micrographs showing EVs from the FSGS and control groups. SigmaPlot software Version 14.0 was used to determine statistical significance between the two groups. ** Represents p<0.001.

Freshly isolated EVs were analyzed by nanoparticle tracking analysis in order to determine the size and concentration of the vesicles in each sample of the FSGS group and the healthy control group. The mean size of the EVs was found to be greater in the FSGS group compared to the control group (Fig 2A). However, there was not a statistically significant difference in concentration between the two groups (Fig 2B). Next, we probed for multiple EV markers including Caveolin-1, CD9, and Syntenin (Fig 2C). A Western blot for HDAC2 was performed as negative control marker. Each sample from the FSGS patient group and the healthy subject control group was probed for Uromodulin (Tamm-Horsfall protein) by Western blot (Fig 2C).in order to normalize the EV concentrations (Table 2). Each EV preparation from the two groups showed enrichment of each of these markers (Fig 2C). In order to further show the general size of EVs present in the two groups are within the typical size range of exosomes and to show the absence of aggregated proteins in the EV preparations, transmission electron microscopy was performed. As shown in the electron micrographs in Fig 2D, the EVs from the two groups were smaller than 150nm in diameter.

**Table 2:** EV concentration normalized to Uromodulin (Tamm-Horsfall protein). FSGS n = 5, Control n = 5; p-value of 0.208.

GROUP	SAMPLE	CONCENTRATION NORMALIZED TO UROMODULIN	MEAN ± SEM NORMALIZED CONCENTRATION	STUDENT’S T-TEST COMPARISON
**FSGS**	FSGS 1 FSGS 2 FSGS 3 FSGS 4 FSGS 5	5.03E+07 5.70E+07 8.41E+07 3.86E+07 9.63E+07	6.53E+07 ± 1.08E+07	FSGS vs. Control
**CONTROL**	CTRL 1 CTRL 2 CTRL 3 CTRL 4 CTRL 5	6.25E+07 1.65E+08 5.40E+07 1.63E+08 6.90E+07	1.03E+08 ± 2.57E+07	

### Urinary EVs from FSGS patients increase STAT3 activation in normal human mesangial cells

**Fig 3:**
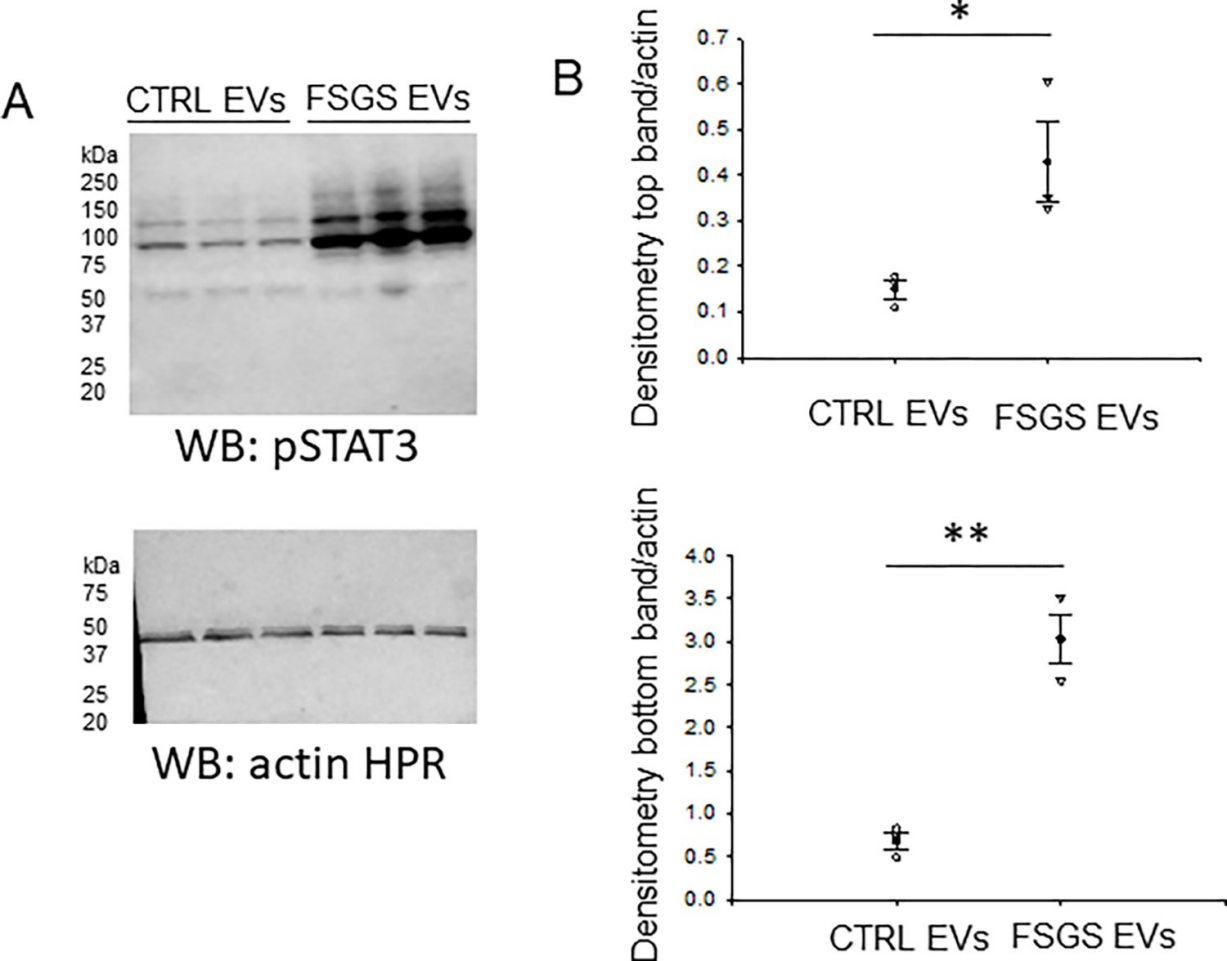
Western blot analysis of phospho-STAT-3 protein after challenging human mesangial cells with EVs from pediatric FSGS patients or age-matched healthy control subjects. **A.** Representative Western blot of phospho-STAT-3 (pSTAT-3) (top blot) and beta actin (bottom blot) after treating human mesangial cells with EVs from FSGS patients or healthy control subjects. The first three lanes represent cellular lysates from three independent experiments of cells treated with control urinary EVs while the last three lanes represent cellular lysates from three independent experiments of cells treated with urinary EVs from FSGS patients. B. Densitometric analysis of the top and bottom immunoreactive bands of the pSTAT-3 blot in panel A normalized to the actin blot. SigmaPlot software Version 14.0 was used to determine statistical significance between the two groups * represents p<0.05 ** represents a p<0.001.

Next, we investigated whether urinary EVs from FSGS patients compared to those of healthy subjects could play a role in the activation of signaling pathways in normal human mesangial cells as part of the disease mechanism. Human mesangial cells were treated for 24 hours with a pool of EVs from five FSGS patients or the same number of samples from the control group. Thereafter, the cells were harvested for protein and the cellular lysates were probed for various targets including the phosphorylated signal transducer and activator of transcription-3 (STAT-3). As shown in Fig 3, human mesangial cells treated with urinary EVs from FSGS patients compared to EVs from control subjects increased phospho-STAT-3 protein expression.

### Urinary EVs from FSGS patients increase expression of the proliferation markers PCNA and ki-67 in normal human mesangial cells

**Fig 4:**
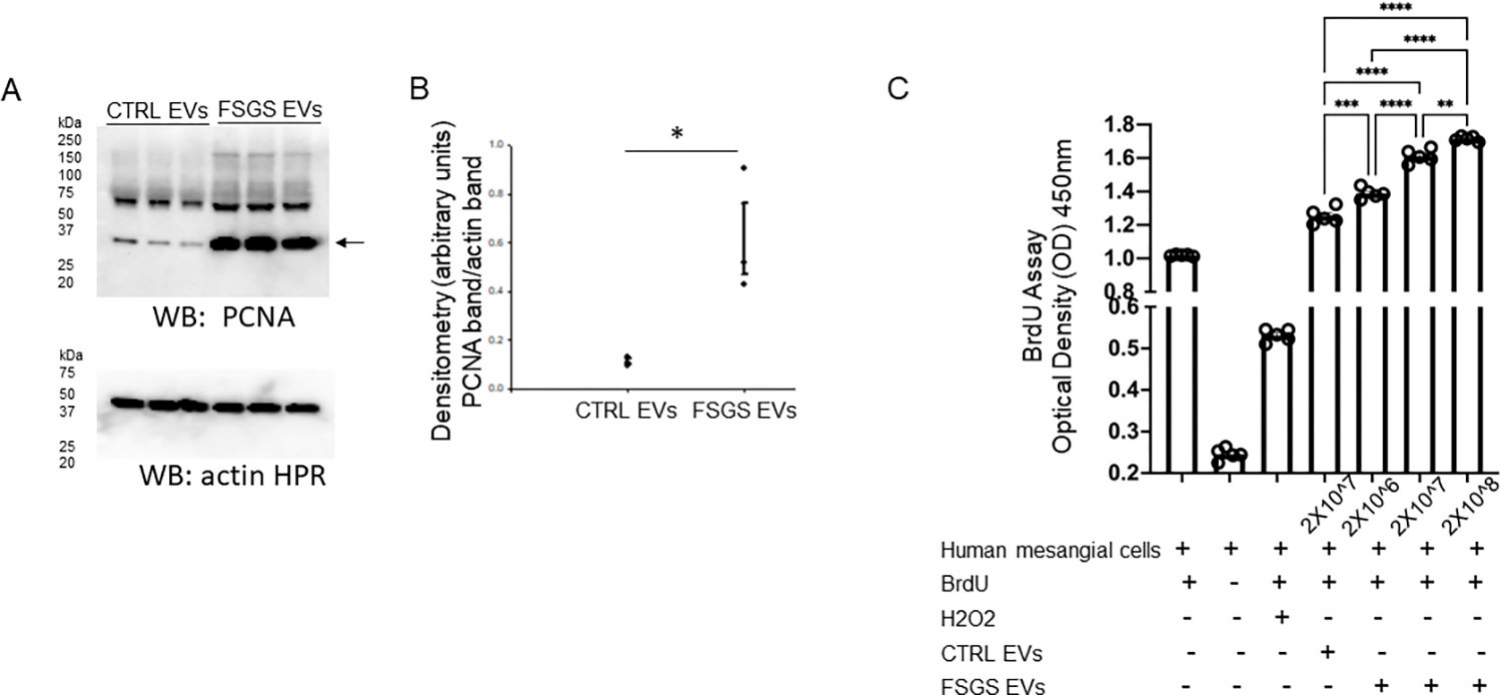
Western blot analysis of PCNA after treating human mesangial cells treated with urinary EVs from FSGS patients or control subjects. **A.** Western blot of PCNA (top blot) and actin (bottom blot). The first three lanes represent cellular lysates from three independent experiments of cells treated with control urinary EVs while the last three lanes represent cellular lysates from three independent experiments of cells treated with urinary EVs from FSGS patients. **B.** Densitometric analysis of the immunoreactive blot in panel A. SigmaPlot software Version 14.0 was used to determine statistical significance between the two groups. * represents a p-value <0.05. **C.** Cell proliferation of human mesangial cells treated with either H2O2, EVs (2X10^7 particles/ml) from control subjects, or EVs (2X10^6, 2X10^7, or 2X10^8 particles/ml) from FSGS patients. Additional controls included treatment of human mesangial cells with or without BrdU. A two-way ANOVA was performed using GraphPad Prism 9 to determine statistical significance between the groups. ** represents a p-value <0.01, *** represents a p-value <0.001, **** represents a p-value<0.0001.

Next, we used specific antibodies against the proliferation marker PCNA to probe for changes in the protein expression of these markers from normal human mesangial cells challenged with EVs from FSGS patients or control subjects. Western blot and densitometric analysis showed PCNA protein expression was greater in the human mesangial cells challenged with the urinary EVs from the FSGS group compared to the control group (Fig 4A and 4B).

Next, to further demonstrate the ability of FSGS EVs to increase proliferation in human mesangial cells we performed a dose dependent BrdU cell proliferation assay. Human mesangial cells were treated with three different concentrations of EVs (2X10^6, 2X10^7, to 2X10^8 particles/mL) from the FSGS group for 6 hours and compared to cells treated with control EVs at a concentration of 2X10^7 for the same period of time. One set of controls included one group of untreated cells being treated with BrdU and another group of untreated cells being treated without BrdU. Another control included another group of cells being treated with BrdU and H2O2. Cells treated with BrdU and FSGS EVs showed increased cellular proliferation in a dose dependent manner compared to each of the internal controls and the experiment control which included cells treated with control EVs (Fig 4C).

**Fig 5:**
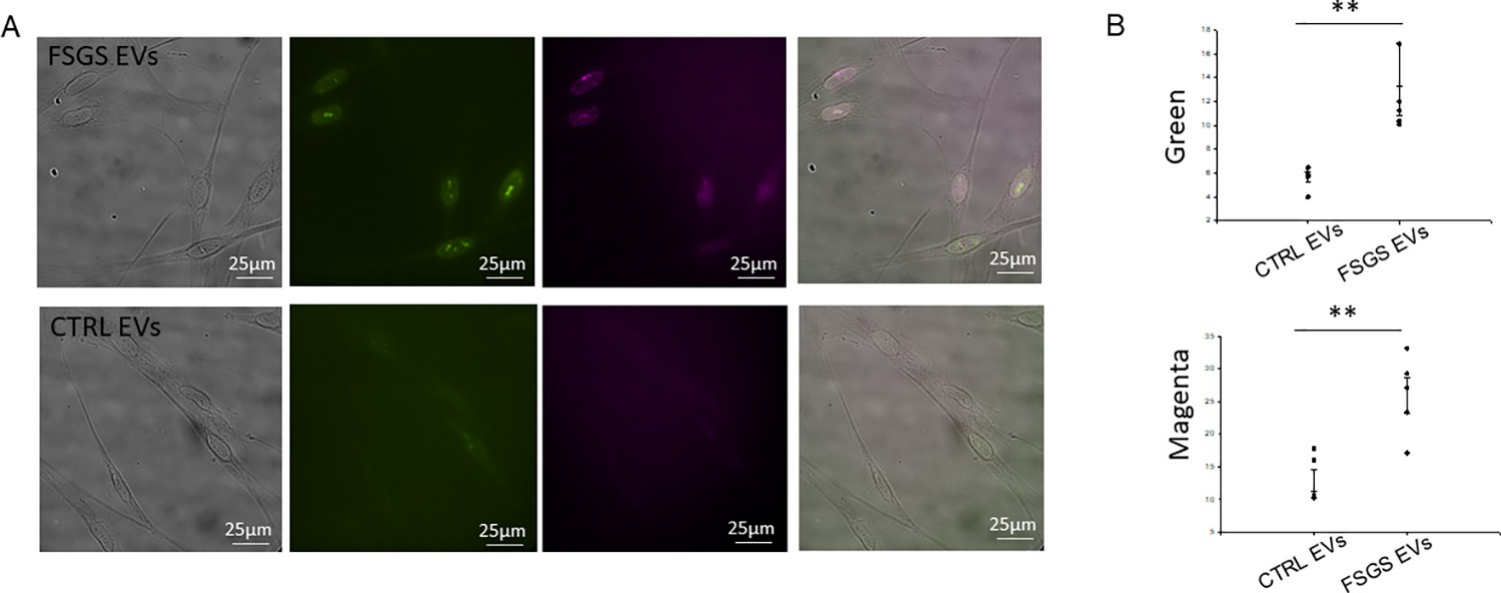
Immunofluorescence microscopy analysis of normal human mesangial cell proliferation after challenging the cells with urinary EVs from pediatric FSGS patients or age-matched healthy control subjects. **A.** Representative images of the proliferative markers ki-67 (green) and PCNA (magenta) in acetone:methanol fixed normal human mesangial cells challenged with urinary EVs from FSGS patients (pool of 5 patients) (top panels) or with urinary EVs from healthy control subjects (pool of 5 subjects) (bottom panels). A 2”x2” box was placed over 5–7 cells and the image was cropped and saved in Tiff format for Image J analysis. B. Densitometric analysis of the ki-67 and PCNA positive staining in panel A. Five cells for each marker were analyzed for each group. SigmaPlot software Version 14.0 was used to determine statistical significance between the two groups ** represents a p<0.001.

In order to corroborate the Western blot results showing urinary EVs from FSGS patients induce proliferation in human mesangial cells and the BrdU cell proliferation experiments, we performed immunofluorescence studies using PCNA specific antibody as well as specific antibody against a second proliferation marker, ki67. As illustrated in Fig 5, urinary EVs from FSGS patients resulted in greater immunofluorescence intensity of both PCNA and ki67 in human mesangial cells compared to the control EVs after being treated for 6 hours (Fig 5).

## Discussion

FSGS is a common cause of CKD and ESKD in adolescents and young adults [12]. The cause of primary FSGS is still unknown. It is thought to be secondary to circulating molecules that lead to podocyte effacement, atrophy/autophagy, and segmental sclerosis [12]. However, the disease process and the interaction between glomerular cells is not fully understood.

Signal transducer and activator of transcription 3 (STAT-3) is a member of the signal transducer and activator of transcription (STAT) protein family [32]. STAT-3 signaling can be activated by a multitude of upstream factors, including cytokines, chemokines, and growth factors [33]. The expression of a variety of genes in response to STAT-3 activation, plays a key role in cell growth by the progression of the cell cycle from G1 to the S phase [34,35]. STAT-3 activation is involved in mesangial cell (MC) proliferation and inactivation of STAT-3 reduces the MC proliferation [36]. It was shown that STAT3 knock out mice show less inflammation and proliferation in glomeruli [37]. In our study we showed that STAT-3 activation drives proliferation of mesangial cells.

Mesangial cell proliferation can be triggered by infections due to increase proinflammatory cytokines such as Il-6, and IL-8 immune complexes (IgA disease and lupus nephritis [37,38]. Mesangial proliferation causes ECM expansion that is critical to progressive glomerular injury and sclerosis. Takahashi et al showed that activation of STAT-3 is a key signaling pathway for mesangial proliferation, ECM expansion and progression to glomerulosclerosis in a glomerulonephritis model [39]. They showed inhibiting the pathway significantly decreased the expression of Col IV in cultured mesangial cells [39]. Since ECM expansion and mesangial proliferation are shown to be key components of glomerulosclerosis, STAT-3 inhibition was investigated for inhibition of glomerulosclerosis in many different kidney diseases such as diabetic or obstructive nephropathy in an animal model [40,41].

Previous studies investigated EV profiles in FSGS. Zhou et al showed exosomal WT-1 levels are higher in pediatric FSGS and steroid sensitive nephrotic syndrome (SSNS) [42]. Lee at all showed only 60% of pediatric urine samples was positive for WT-1 and there was no significant difference between FSGS and healthy controls [43]. Zhang et al investigated the plasma miRNA profile of FSGS patients and compared it with that of healthy controls. They found plasma miR-186 correlated with the degree of proteinuria in patients with FSGS [44]. To our knowledge, no study has evaluated the effects of EVs on STAT-3 activation in glomerular cells.

In our study, we collected urine samples from healthy control subjects and FSGS patients who were in partial remission under angiotensinogen converting enzyme inhibitors (ACEi) and Rituximab due to steroid resistance. We compared both groups based on demographics, blood work, and urine analysis. The only difference between the groups was the mean serum creatinine and serum chloride was significantly higher in the FSGS group. (Table 1). It is expected to see higher creatinine and higher chloride levels in the FSGS group due to glomerular damage, having a lower GFR, the use of ACE inhibitors, or being on a sodium restricted diet. On the other hand, all FSGS patients included in this study were on ACE inhibitors and rituximab. Further studies are needed to evaluate the effect of rituximab on EVs cargo sorting and release.

Podocytopathy is the common pathology of primary FSGS [45]. However, it is still not clear what causes podocyte injury. We hypothesis that the molecules that are carried by EVs activate STAT-3 and cause increased mesangial cell proliferation. We speculate that EVs that originate from various cell types including podocytes cause mesangial proliferation and secondary changes in the glomeruli.

This is the first study that evaluates the effects of EVs isolated from FSGS pediatric patients compared to healthy control subjects on normal human mesangial cells. In this study we investigated the molecular mechanism by which EVs from FSGS patients induce proliferation of normal human mesangial cells. We showed EVs from the FSGS group activates the STAT-3 pathway which presumably leads to the increase in proliferation in a dose dependent manner. The length of time the human mesangial cells were treated with EVs and the concentration of EVs used in our studies are likely both overestimations that native cells would be exposed to *in vivo*. It is difficult to accurately estimate the duration and concentration of EVs that a particular cell type would be exposed to in a physiological or pathophysiological scenario because EV release and uptake are both dynamic processes that are regulated by a myriad of mechanisms.

Mechanistic data show EVs modulate leukocyte adhesion, differentiation and vascular function in inflammation and these studies have greatly improved our understanding of these pathophysiologic processes [46,47]. Experimental results suggest a number of mechanisms are regulated by the release of EVs from one cell type and uptake of EV by similar cells or different cell types [8,48–51]. In the context of the kidney, EVs may promote cross-talk between various cell types and inhibit or activate signaling pathways within different cell types [52,53].

The main limitation of our study was that we had a small sample size of pediatric FSGS patients and healthy control subjects which could contribute to large SD values. It may be possible to collaborate with other institutions in order to recruit more patients to our study and further investigate the role of EVs in the pathophysiology of pediatric FSGS. A second limitation of our study is that our transmission electron microscopy images were not taken on a wide enough field and did not include a dense absorption of EVs on the grids to rule out the possibility of aggregated proteins being present or that our EV preparations could contain a mixed population of EVs including microvesicles in addition to exosomes. Another limitation of the study is that we did not investigate the molecular content of the EVs in the FSGS or control group. Future follow-up studies will include mass spectrometry based lipidomics and proteomics to identify key lipids and proteins that are upregulated or downregulated between the two groups.

Taken together, we showed that urinary EVs from pediatric FSGS patients are larger in size when compared to that of healthy controls, and the cargo within the EVs between the two groups is likely unique. Our data suggest the cargo within FSGS EVs increase mesangial cell proliferation which may in part be due to activation of the STAT-3 pathway.

## Supporting information

S1 File (PDF)

S1 Raw images (PDF)
