# Supplementary material for: Extracellular vesicles from focal segmental glomerulosclerosis pediatric patients induce STAT3 activation and mesangial cell proliferation
Source: PLoS One. 2022 Nov 14;17(11):e0274598. doi: 10.1371/journal.pone.0274598 (PMC9662727; doi:10.1371/journal.pone.0274598)
Supplement: S1 File — (PDF) [file pone.0274598.s001.pdf]

S1

| FSGS=1. Control=2 | Specimen | subgroup: P | CNS | type of CN | gender M | ethnicity: | age at coll | Wt=kg | ht=cm | UA-osm | Ua-Na | Ua-K | Ua- Cl | ser-Cre | se-BUN | ser-album | ser-Na | ser-K | ser-Cl | ta |
|---|---|---|---|---|---|---|---|---|---|---|---|---|---|---|---|---|---|---|---|---|
| 1 | 4 | 3 | 1 | 4 | 1 | 3 | 9 | 30.7 | 137.4 | 933 | 6 | 27.6 | 33 | 4.37 | 25 | 3.7 | 138 | 4.1 | 99 | |
| 1 | 7 | 3 | 1 | 4 | 1 | 1 | 19 | 70 | 166.5 | 341 | 20 | 9.5 | 31 | 0.52 | 19.2 | 4 | 140 | 3.4 | 106 | |
| 1 | 8 | 3 | 1 | 4 | 1 | 1 | 15 | 109.1 | 157.8 | 1308 | 105 | 72.9 | 149 | 0.67 | 9 | 3.8 | 139 | 4 | 106 | |
| 1 | 11 | 3 | 1 | 4 | 2 | 2 | 13 | 36.7 | 152.5 | 1099 | 308 | 44.9 | 336 | 0.35 | 12 | 4.6 | 138 | 3.9 | 106 | |
| 1 | 15 | 3 | 1 | 1 | 1 | 2 | 21 | 72 | 156 | 230 | 103 | 7.2 | 107 | 1.23 | 9 | 4.2 | 137 | 3.2 | 109 | |
| 2 | 17 | 4 | 0 | 0 | 2 | 3 | 6 | 35.5 | 124.5 | 441 | 92 | 27 | 93 | 0.4 | 10 | | 139 | 4.1 | 104 | |
| 2 | 19 | 4 | 0 | 0 | 1 | 3 | 15 | 48 | 152.4 | 948 | 166 | 62.1 | 159 | | | | | | | |
| 2 | 20 | 4 | 0 | 0 | 1 | 3 | 11 | 49 | 149.9 | 825 | 156 | 24.5 | 155 | | | | | | | |
| 2 | 21 | 4 | 0 | 0 | 1 | 1 | 17 | 47.1 | 160.4 | 395 | 120 | 6.6 | 124 | 0.5 | 16 | 4 | 139 | 4.7 | 103 | |
| 2 | 22 | 4 | 0 | 0 | 1 | 2 | 7 | 29 | 131.5 | 1114 | 163 | 26.4 | 135 | 0.24 | 8 | 4.2 | 139 | 4.3 | 105 | |

S2

| 6-FSGS | 7 | 8-Controls | 9 | 10 | 11 | 12-size | 13-mean | 14-SE | 15-SEM |
|---|---|---|---|---|---|---|---|---|---|
| C4 | 93.2000 | C16 | 82.7000 | | FSGS vs CC | 5.0000 | 106.0400 | 7.3990 | 7.3990 |
| C7 | 93.6000 | C17 | 76.0000 | | | 7.0000 | 79.4140 | 7.7320 | 7.7320 |
| C8 | 130.8000 | C18 | 84.4000 | | | | | | |
| C11 | 115.3000 | C19 | 68.3000 | | | | | | |
| C15 | 97.3000 | C20 | 89.2000 | | | | | | |
| | | C21 | 44.2000 | | | | | | |
| | | C22 | 111.1000 | | | | | | |

S3

| SAMPLE | CONCENTRATION NORMALIZED TO UROMODULIN | MEAN ± SEM NORMALIZED CONCENTRATION |
|---|---|---|
| FSGS 1 | 5.03E+07 | 6.53E+07 ± 1.08E+07 |
| FSGS 2 | 5.70E+07 | |
| FSGS 3 | 8.41E+07 | |
| FSGS 4 | 3.86E+07 | |
| FSGS 5 | 9.63E+07 | |
| CTRL 1 | 6.25E+07 | 1.03E+08 ± 2.57E+07 |
| CTRL 2 | 1.65E+08 | |
| CTRL 3 | 5.40E+07 | |
| CTRL 4 | 1.63E+08 | |
| CTRL 5 | 6.90E+07 | |

S4

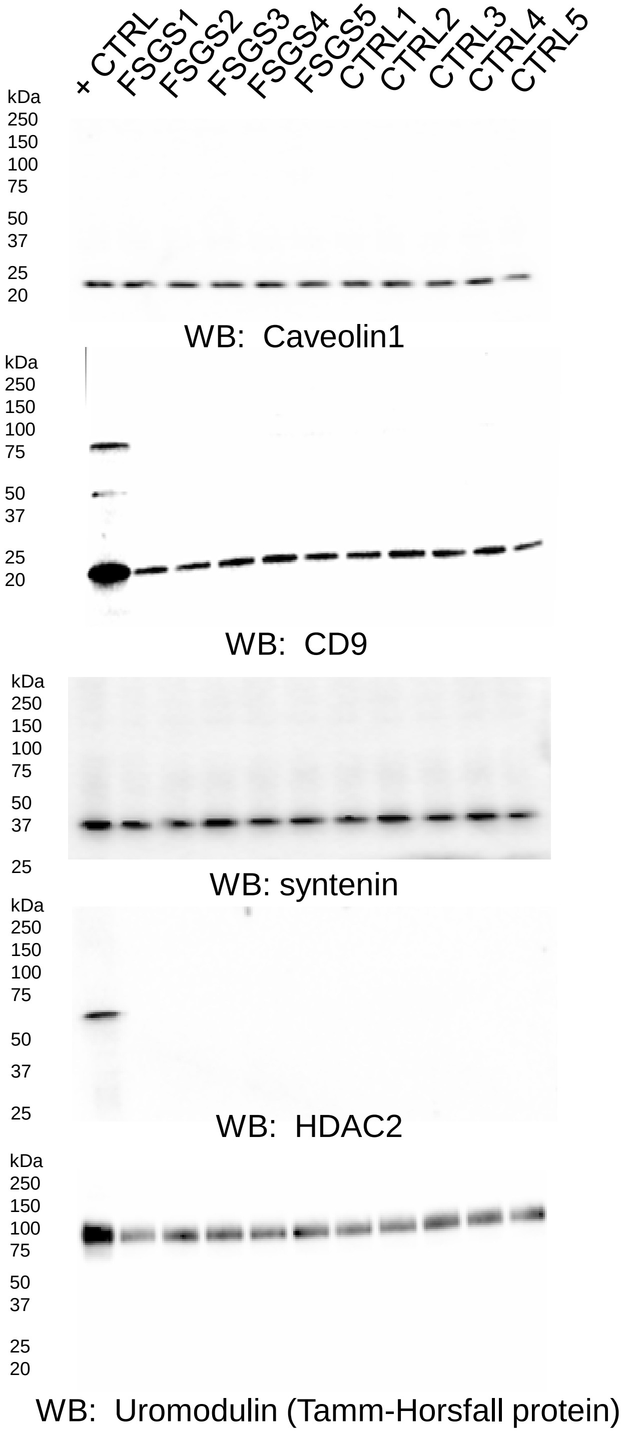
+ CTRL
FSGS1
FSGS2
FSGS3
FSGS4
FSGS5
CTRL1
CTRL2
CTRL3
CTRL4
CTRL5
kDa
250
150
100
75
50
37
25
20
WB: Caveolin1
WB: CD9
WB: syntenin
WB: HDAC2
WB: Uromodulin (Tamm-Horsfall protein)

S5

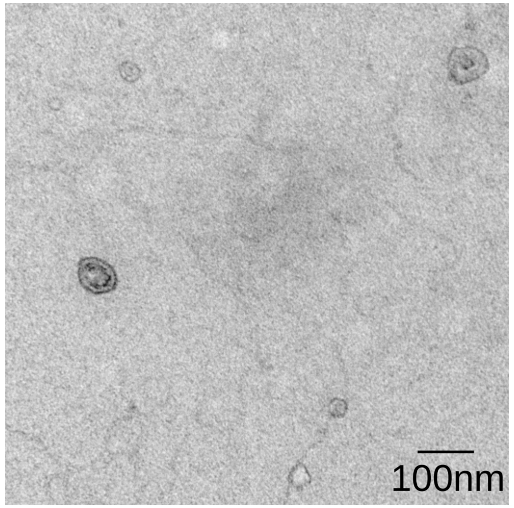


FSGS

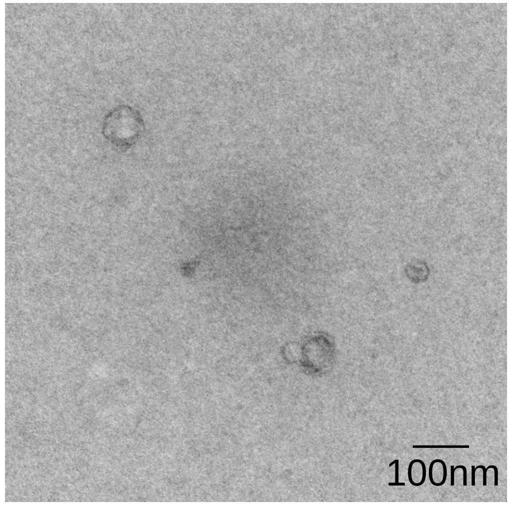


Control

S6

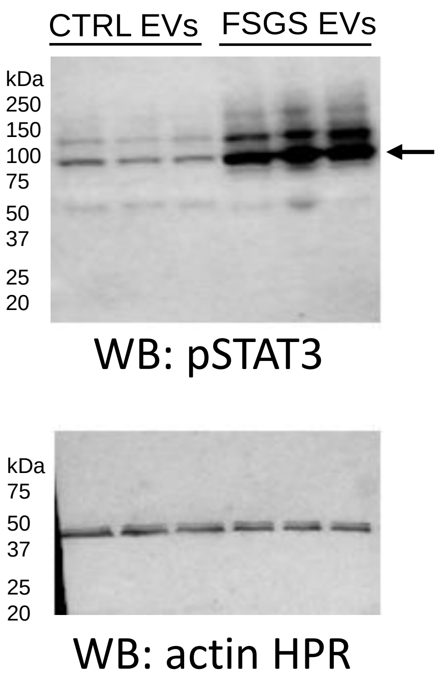

CTRL EVs
FSGS EVs
kDa
250
150
100
75
50
37
25
20
WB: pSTAT3
kDa
75
50
37
25
20
WB: actin HPR

S7

| | 1 | 2-ctrl | 3-FSGS | 4 | 5-size | 6-mean | 7-SE | 8-SEM | 9 | 10-ctrl | 11-FSGS | 12 | 13-size | 14-mean | 15-SE | 16-SEM | 17 |
|---|---|---|---|---|---|---|---|---|---|---|---|---|---|---|---|---|---|
| 1 | Naile | 0.1650 | 0.6055 | | 3.0000 | 0.1510 | 0.0210 | 0.0210 | | 0.7330 | 3.5172 | | 3.0000 | 0.6800 | 0.0994 | 0.0994 | |
| 2 | WB | 0.1094 | 0.3281 | | 3.0000 | 0.4280 | 0.0888 | 0.0888 | | 0.4870 | 2.5449 | | 3.0000 | 3.0260 | 0.2810 | 0.2810 | |
| 3 | pSTAT3 | 0.1780 | 0.3516 | | | | | | | 0.8186 | 3.0172 | | | | | | |
| 4 | 125.0000 | | | | | | | | 85.0000 | | | | | | | | |
| 5 | | | | | | | | | | | | | | | | | |
| 6 | | | | | | | | | | | | | | | | | |

S8

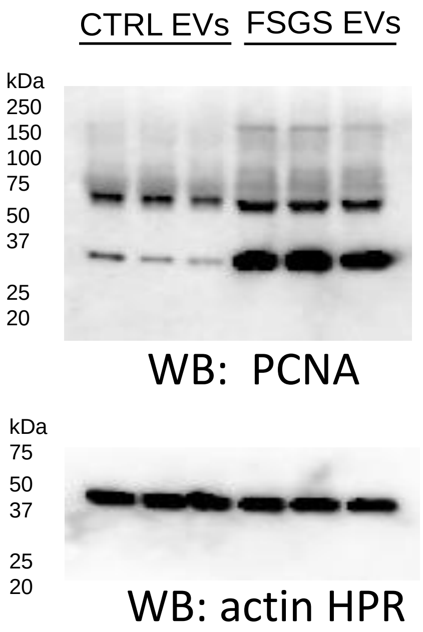
CTRL EVs
FSGS EVs
kDa
250
150
100
75
50
37
25
20
WB: PCNA
kDa
75
50
37
25
20
WB: actin HPR

S9

| 1 | 2-CTRL | 3-PCNA | 4 | 5 | 6 | 7 | 8 | 9 | 10 | 11 |
|---|---|---|---|---|---|---|---|---|---|---|
| PCNA | 0.1335 | 0.5202 | | Column | Size | Missing | Mean | Std Dev | Std. Error | C.I. of Mea |
| | 0.1073 | 0.4295 | | CTRL | 3.0000 | 0.0000 | 0.1120 | 0.0202 | 0.0116 | 0.0501 |
| | 0.0938 | 0.9027 | | PCNA | 3.0000 | 0.0000 | 0.6170 | 0.2510 | 0.1450 | 0.6240 |
| | | | | | | | | | | |

# S10

| 10K hmesang cells+BrdU | 10K hmesang cells-BrdU | 10K hmesang cells +brdU+H2O2 | 10K hmesang cells +BrdU+Ctrl EVs 2X10^7 | 10K hmesang cells+BrdU+FSGS EVs 2X10^6 | 10K hmesang cells+BrdU+FSGS EVs 2X10^7 | 10K hmesang cells+BrdU+FSGS EVs 2X10^8 |
|---|---|---|---|---|---|---|
| 1.0239 | 0.2248 | 0.5102 | 1.2376 | 1.3968 | 1.5579 | 1.7275 |
| 1.0146 | 0.2632 | 0.5329 | 1.3237 | 1.4363 | 1.6373 | 1.7072 |
| 1.0222 | 0.2429 | 0.5453 | 1.2743 | 1.3854 | 1.6638 | 1.6952 |
| 1.0117 | 0.2444 | 0.5224 | 1.2249 | 1.3507 | 1.5932 | 1.7308 |
| 1.0194 | 0.2529 | 0.5448 | 1.2031 | 1.3737 | 1.6061 | 1.7164 |

FSGS EVs

CTRL EVs

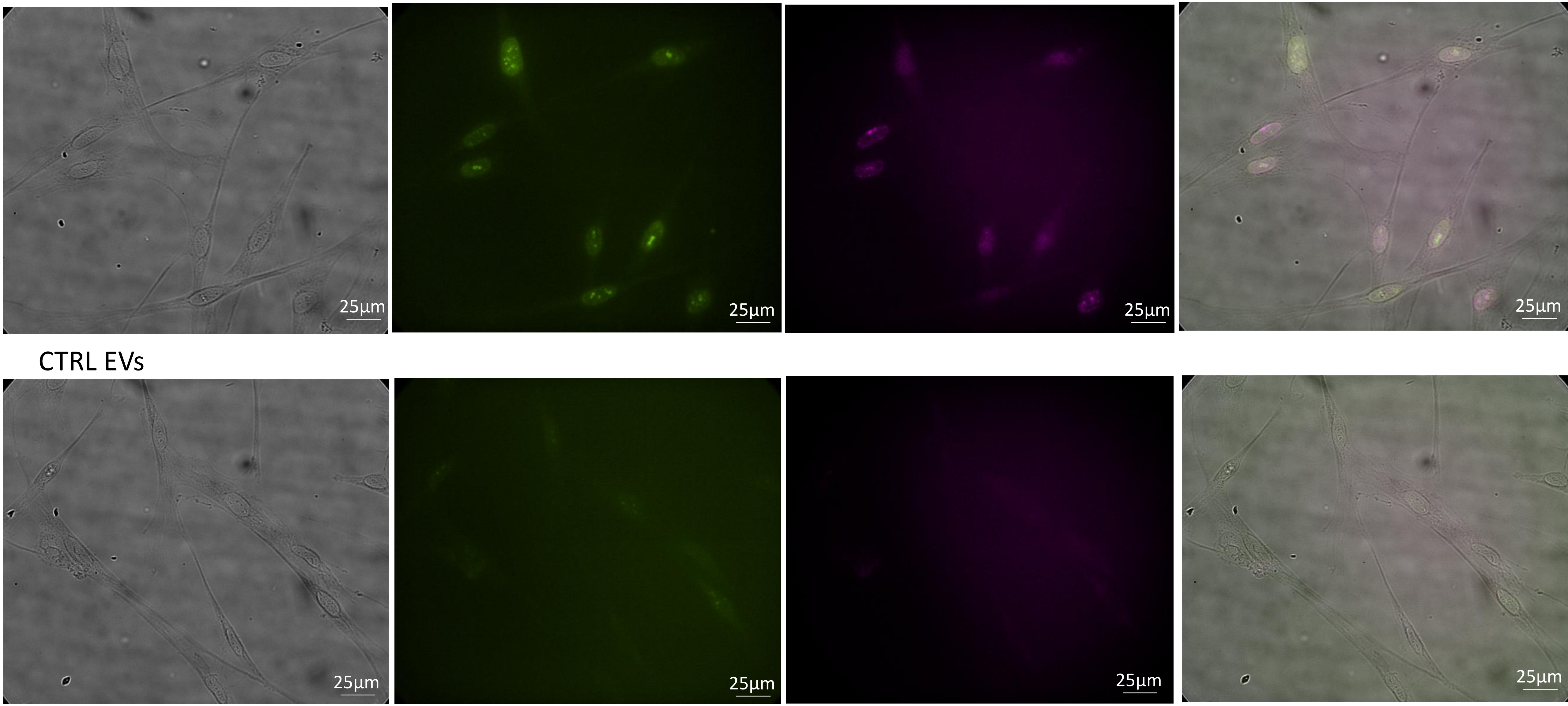

S12

| FSGS Green | FSGS Magenta | | CTRL Green | FSGS Magenta | |
|---|---|---|---|---|---|
| 16.833 | 27.043 | | 6.438 | 10.196 | |
| 11.981 | 23.298 | | 6.443 | 17.77 | |
| 10.335 | 29.272 | | 5.883 | 10.227 | |
| 10.072 | 33.081 | | 5.695 | 16.047 | |
| 11.214 | 17.079 | | 3.98 | 10.564 | |
