# Supplementary material for: Extracellular vesicles from focal segmental glomerulosclerosis pediatric patients induce STAT3 activation and mesangial cell proliferation
Source: PLoS One. 2022 Nov 14;17(11):e0274598. doi: 10.1371/journal.pone.0274598 (PMC9662727; doi:10.1371/journal.pone.0274598)
Supplement: S1 Raw images — (PDF) [file pone.0274598.s002.pdf]

S1

FSGS=1. Control=2	Specimen	subgroup: P	CNS	type of CN	gender M	ethnicity:	age at coll	Wt=kg	ht=cm	UA-osm	Ua-Na	Ua-K	Ua- Cl	ser-Cre	se-BUN	ser-album	ser-Na	ser-K	ser-Cl	ta
1	4	3	1	4	1	3	9	30.7	137.4	933	6	27.6	33	4.37	25	3.7	138	4.1	99	
1	7	3	1	4	1	1	19	70	166.5	341	20	9.5	31	0.52	19.2	4	140	3.4	106	
1	8	3	1	4	1	1	15	109.1	157.8	1308	105	72.9	149	0.67	9	3.8	139	4	106	
1	11	3	1	4	2	2	13	36.7	152.5	1099	308	44.9	336	0.35	12	4.6	138	3.9	106	
1	15	3	1	1	1	2	21	72	156	230	103	7.2	107	1.23	9	4.2	137	3.2	109	
2	17	4	0	0	2	3	6	35.5	124.5	441	92	27	93	0.4	10		139	4.1	104	
2	19	4	0	0	1	3	15	48	152.4	948	166	62.1	159							
2	20	4	0	0	1	3	11	49	149.9	825	156	24.5	155							
2	21	4	0	0	1	1	17	47.1	160.4	395	120	6.6	124	0.5	16	4	139	4.7	103	
2	22	4	0	0	1	2	7	29	131.5	1114	163	26.4	135	0.24	8	4.2	139	4.3	105	

S2

6-FSGS	7	8-Controls	9	10	11	12-size	13-mean	14-SE	15-SEM
C4	93.2000	C16	82.7000		FSGS vs CC	5.0000	106.0400	7.3990	7.3990
C7	93.6000	C17	76.0000			7.0000	79.4140	7.7320	7.7320
C8	130.8000	C18	84.4000						
C11	115.3000	C19	68.3000						
C15	97.3000	C20	89.2000						
		C21	44.2000						
		C22	111.1000						

S3

SAMPLE	CONCENTRATION NORMALIZED TO UROMODULIN	MEAN ± SEM NORMALIZED CONCENTRATION
FSGS 1	5.03E+07	6.53E+07 ± 1.08E+07
FSGS 2	5.70E+07	
FSGS 3	8.41E+07	
FSGS 4	3.86E+07	
FSGS 5	9.63E+07	
CTRL 1	6.25E+07	1.03E+08 ± 2.57E+07
CTRL 2	1.65E+08	
CTRL 3	5.40E+07	
CTRL 4	1.63E+08	
CTRL 5	6.90E+07	

S4

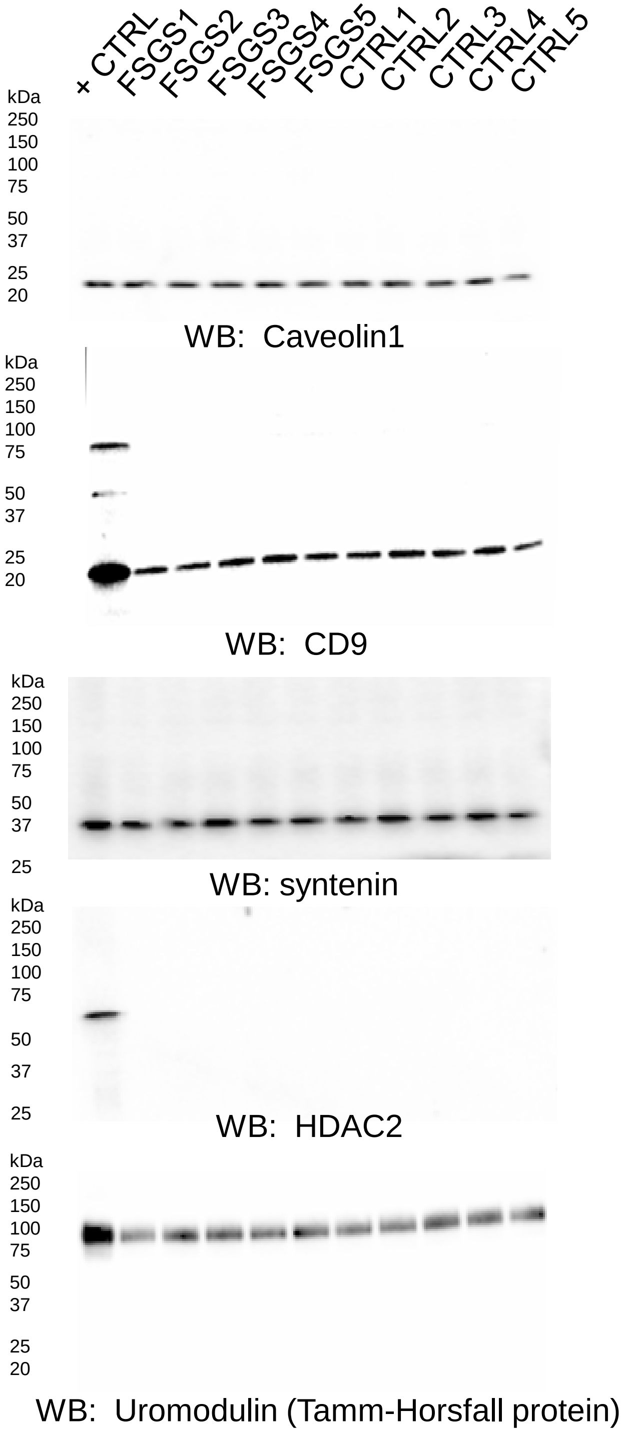
+ CTRL
FSGS1
FSGS2
FSGS3
FSGS4
FSGS5
CTRL1
CTRL2
CTRL3
CTRL4
CTRL5
kDa
250
150
100
75
50
37
25
20
WB: Caveolin1
WB: CD9
WB: syntenin
WB: HDAC2
WB: Uromodulin (Tamm-Horsfall protein)

S5

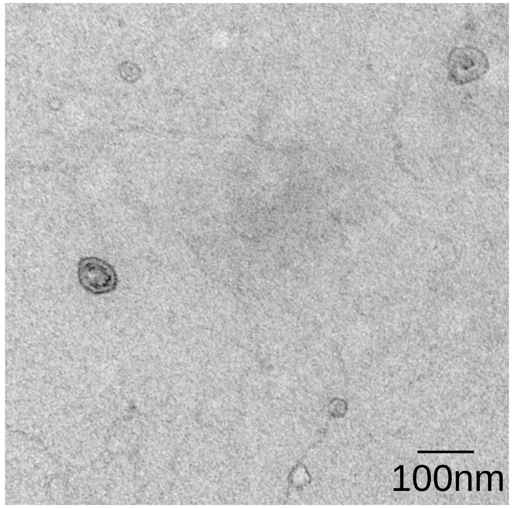

FSGS

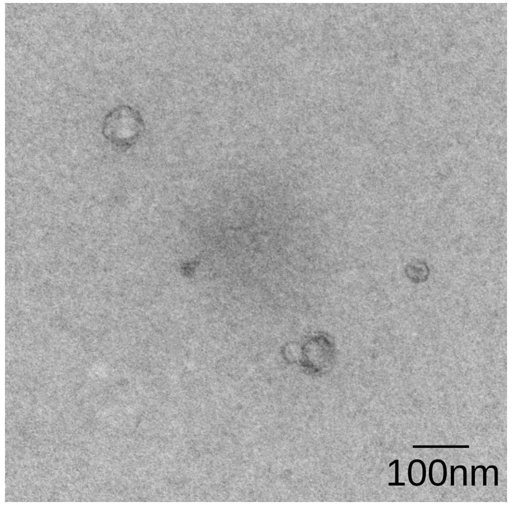

Control

S6

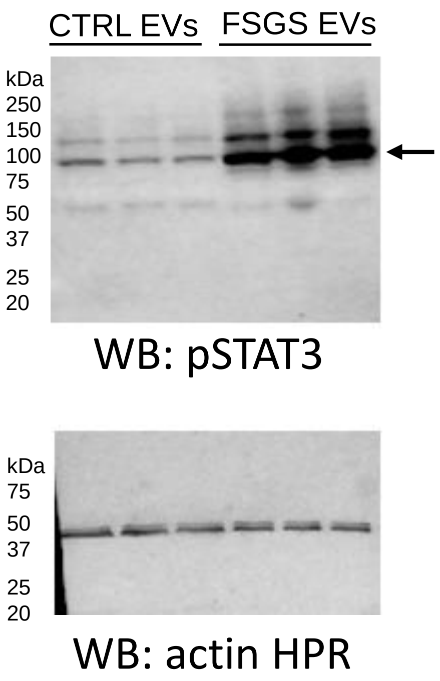
CTRL EVs
FSGS EVs
kDa
250
150
100
75
50
37
25
20
WB: pSTAT3
kDa
75
50
37
25
20
WB: actin HPR

S7

	1	2-ctrl	3-FSGS	4	5-size	6-mean	7-SE	8-SEM	9	10-ctrl	11-FSGS	12	13-size	14-mean	15-SE	16-SEM	17
1	Naile	0.1650	0.6055		3.0000	0.1510	0.0210	0.0210		0.7330	3.5172		3.0000	0.6800	0.0994	0.0994	
2	WB	0.1094	0.3281		3.0000	0.4280	0.0888	0.0888		0.4870	2.5449		3.0000	3.0260	0.2810	0.2810	
3	pSTAT3	0.1780	0.3516							0.8186	3.0172						
4	125.0000								85.0000								
5																	
6																	

S8

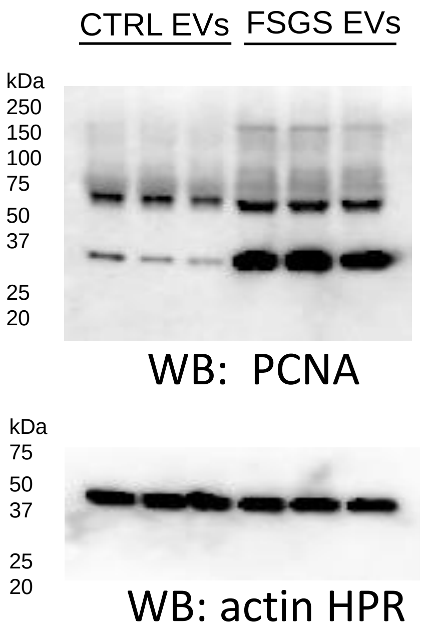
CTRL EVs
FSGS EVs
kDa
250
150
100
75
50
37
25
20
WB: PCNA
kDa
75
50
37
25
20
WB: actin HPR

S9

1	2-CTRL	3-PCNA	4	5	6	7	8	9	10	11
PCNA	0.1335	0.5202		Column	Size	Missing	Mean	Std Dev	Std. Error	C.I. of Mea
	0.1073	0.4295		CTRL	3.0000	0.0000	0.1120	0.0202	0.0116	0.0501
	0.0938	0.9027		PCNA	3.0000	0.0000	0.6170	0.2510	0.1450	0.6240

S10

10K hmesang cells+BrdU	10K hmesang cells-BrdU	10K hmesang cells +brdU+H2O2	10K hmesang cells +BrdU+Ctrl EVs 2X10^7	10K hmesang cells+BrdU+FSGS EVs 2X10^6	10K hmesang cells+BrdU+FSGS EVs 2X10^7	10K hmesang cells+BrdU+FSGS EVs 2X10^8
1.0239	0.2248	0.5102	1.2376	1.3968	1.5579	1.7275
1.0146	0.2632	0.5329	1.3237	1.4363	1.6373	1.7072
1.0222	0.2429	0.5453	1.2743	1.3854	1.6638	1.6952
1.0117	0.2444	0.5224	1.2249	1.3507	1.5932	1.7308
1.0194	0.2529	0.5448	1.2031	1.3737	1.6061	1.7164

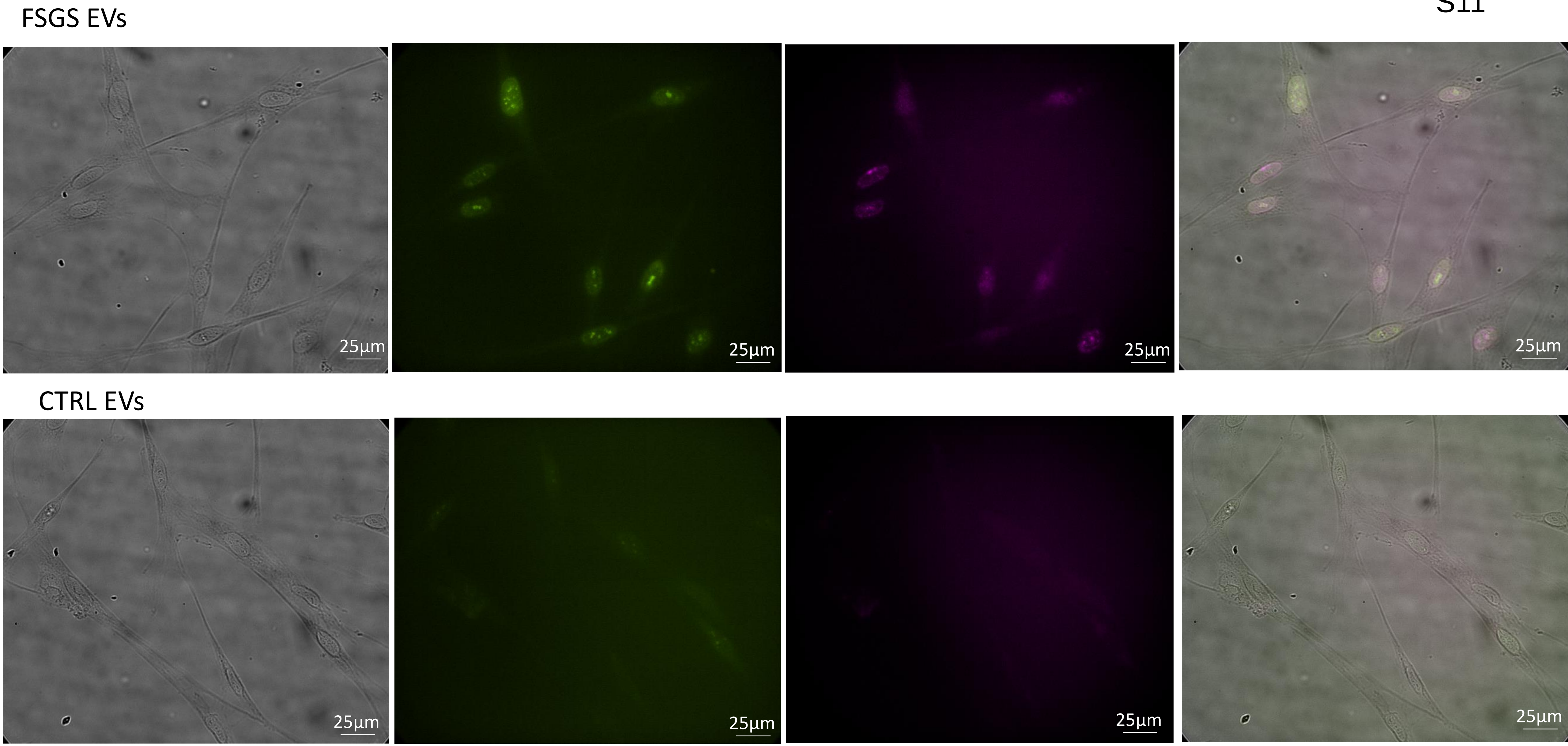
FSGS EVs
25µm
25µm
25µm
25µm
CTRL EVs
25µm
25µm
25µm
25µm

S12

FSGS Green	FSGS Magenta		CTRL Green	FSGS Magenta	
16.833	27.043		6.438	10.196	
11.981	23.298		6.443	17.77	
10.335	29.272		5.883	10.227	
10.072	33.081		5.695	16.047	
11.214	17.079		3.98	10.564	
